# The Chancellor to Confer with the Greater Hospitals

**Published:** 1912-12-14

**Authors:** 


### The Hospital

The Workers' Newspaper of Administrative Medicine and Institutional Life, Administration, National Insurance and Health.

No. 1380, Vol. LIII. SATURDAY, DECEMBER 14, 1912. PRICE ONE PENNY.

### THE CHANCELLOR TO CONFER WITH THE GREATER HOSPITALS.

We have satisfaction in announcing that the Chancellor of the Exchequer has decided to invite representatives of a few of the chief voluntary hospitals to confer with him on the questions we have been labouring in these columns for several weeks past. They, of course, relate to the urgent need for an adequate provision of hospital accommodation and treatment for insured persons under the Insurance Act. It is of the first importance that all who may attend this conference shall realise fully that, the crisis which has to be met and faced by the voluntary hospitals is one of momentous gravity. The first safeguard for the voluntary hospitals is to secure, before January 15 next, that, every possible risk of loss of revenue, from the withdrawal of voluntary contributions by those masters and others, who have compulsorily to pay for medical and sick benefit under this Act for the persons they may employ, shall be avoided by a businesslike arrangement between the Government and the voluntary hospitals, whereby the voluntary principle may be preserved and strengthened.

We have fully set forth a plan of *pro rata* payments plus 10 per cent., which may well form the basis for a practical arrangement, between the Chancellor and the hospitals, to tide over the difficulty, so as to prevent any serious loss of revenue to these institutions. The Chancellor of the Exchequer, should the National Insurance Act result, as it may result if great care is not exercised, in the loss of something like 50 per cent. of the present revenues of the voluntary hospitals in this country, may find himself in the position of having to find many millions a year in order to secure the hospital provision, which it is essential insured persons shall have ready for their use, whenever they may need it. Once this revenue is lost, it can never be recovered from the same source, a fact which we commend to the most earnest consideration of the Government as well as of the hospital managers. There is not the slightest need or justification for running any such risk, for the Chancellor may, as a prudent statesman, properly make provision by arrangement with the hospitals to underwrite on a *pro rata* basis all the risk which may follow the admission of insured persons as free cases, in the absence of a businesslike arrangement between the Insurance Commissioners or the approved societies and the voluntary hospitals. We do therefore hope that those who meet the Chancellor will keep this point steadily in view, and enforce it with all their might.

No one to-day can forecast exactly what precisely would follow the admission to voluntary hospitals, without question, of every insured person who demands free in-patient treatment after January 15, 1913. What is known is, the most careful calculations show that, should the National Insurance Act as a direct result of permitting the position to grow increasingly complicated and disastrous by a policy of drift, there can be little question that the whole of our hospital system must be changed for the worst. Then we may speedily find ourselves in a position where the State, or the municipalities, or both, as in Germany, have to take over the hospitals at a cost which cannot ultimately be less in the whole than an added charge to the tax- and rate-payers of quite £20,000,000 a year.* Here, then, is a reason which must and does impress members of the House of Commons of all parties and of the present Government with the strong conviction that, a solution must be found, and that the way to find it is by wise counsel in conference at the present juncture, between the Chancellor of the Exchequer, the Insurance Commissioners, and the voluntary hospital authorities.

There are, of course, many matters relating to this question which will have to be determined. These matters include the action of the medical profession, and the attitude which may be taken by the working classes who have heretofore been such liberal supporters of many of the voluntary hospitals. The medical difficulty would be overcome if a basis of *pro rata* payments plus 10 per cent. for medical treatment for all insured persons admitted to voluntary hospitals could be agreed to, even as a tentative measure, between the Chancellor

* *Vide* Evidence before Poor Law Commission.

and the hospital authorities. Under such a system there would be no question of State control, because the obligation would be not on the part of the hospitals, but on the part of the people and Government of this country, for the generous co-operation which the voluntary hospitals would thus extend to the Insurance Commissioners under the Insurance Act. This co-operation would remove the risk of the grave disasters which may follow to a multitude of workers, should the necessary hospital provision not be forthcoming for insured persons, as and when occasion arise. No one who understands the possible consequences to the patients, let alone the increased expenditure and a multitude of other undesirable things which would follow from such a system, would substitute State hospitals for voluntary hospitals in this country. The Government, if they wished, might properly ask that arrangements should be made for the inspection of the wards in which the insured persons were treated on a *pro rata* basis. No hospital management could object to such an inspection, as it would neither carry nor imply any interference whatever with the free administration of these institutions, which have been conducted on the whole with such marked ability and enterprise, especially during the last two decades. What is needed at the moment is wisdom in counsel, a keen spirit of apprehension of the crisis which has arisen, and an earnest desire and stern determination on the part of everybody concerned to find the best way out of the difficulties which have to be faced at the moment. Neither the Government nor the hospitals are in a position to do otherwise than heartily co-operate with each other in the best interests of the sick and suffering. All must have adequate medical and surgical treatment in hospitals. The public at large who in all the circumstances realise, as the overwhelming majority of the members of Parliament agree, that a way must be found to perpetuate and strengthen the voluntary hospitals of this country.

